# Effects of xylitol chewing gum and candies on the accumulation of dental plaque: a systematic review

**DOI:** 10.1007/s00784-021-04225-8

**Published:** 2021-10-22

**Authors:** Eva Söderling, Kaisu Pienihäkkinen

**Affiliations:** Institute of Dentistry, University of Turku, Lemminkäisenkatu 2, 20520 Turku, Finland

**Keywords:** Xylitol, Sorbitol, Maltitol, Chewing gum, Dental plaque, Dental biofilm

## Abstract

**Objectives:**

A systematic review of published data was conducted with the aim of assessing the effects of xylitol consumption on the amount of dental plaque.

**Materials and methods:**

Electronic and hand searches were performed to find clinical studies concerning the effects of xylitol chewing gum or candies on dental plaque. Prospective randomized controlled clinical trials published between 1971 and 2020 conducted in healthy subjects were included in the review.

**Results:**

The initial search identified 424 xylitol articles. After applying inclusion and exclusion criteria, altogether 14 articles (16 studies) were reviewed. The review identified 12 of the total of 14 xylitol chewing gum studies as having fair or high quality. In 13 of the 14 chewing gum studies, xylitol gum decreased plaque accumulation. In six studies, xylitol gum chewing decreased plaque compared to sorbitol gum, and in three studies compared to gum base/no gum. In three fair-quality studies conducted with xylitol candies, plaque accumulation did not change.

**Conclusions:**

Habitual xylitol gum chewing appears to show plaque-reducing effects that differ from those of sorbitol gum. This suggests specific effects for xylitol on plaque accumulation. Xylitol candies appear not to decrease plaque. The heterogeneity of the studies warrants further research.

**Clinical relevance:**

Habitual xylitol gum chewing is likely to decrease plaque.

## Introduction

Dental caries is a lifestyle-related disease, poor oral hygiene and poor dietary habits playing a key role. Caries is initially reversible and can be halted by removing enough of dental biofilm, i.e. dental plaque [1]. Dental biofilm is also a risk factor of periodontal disease. Gingival inflammation in response to dental plaque accumulation is considered a key factor for the onset of periodontitis [2]. Several intrinsic and extrinsic factors, such as saliva and frequent carbohydrate consumption, influence plaque accumulation [3]. These “disease drivers” are also crucial for symbiosis/dysbiosis of the oral microbiota. In recent years, research has focused on ways to increase resistance of the microbiota to dysbiosis [4, 5]. However, plaque accumulation by itself increases the risk of dental disease.

Xylitol is a naturally occurring five-carbon polyol sweetener that appears to have specific, beneficial effects on oral health but also other health benefits [6]. Habitual consumption of xylitol is suggested to reduce caries occurrence [7]. Systematic reviews on the caries-preventive effect of xylitol have nevertheless resulted in varying outcomes [7–9]. The “chewing effect” is considered by some authors to explain the caries-preventive effects of xylitol chewing gum [10, 11]. However, xylitol administered with pastilles [12], syrup [13] and wipes [14] has also reduced caries. There is good evidence that habitual xylitol consumption decreases counts of caries-associated mutans streptococci [15]. Xylitol appears to act as an oral prebiotic decreasing mutans streptococci without affecting the overall microbiota, and may thus increase the resistance of the microbiota to dysbiosis [15].

Habitual xylitol consumption has been associated with a decrease in the amount of dental plaque [16], though studies that do not support this result have also been published [11]. Habitual, long-term xylitol consumers were reported to have low levels of dental plaque compared to non-consumers of xylitol [17]. It has been suggested that the plaque of xylitol users is less adhesive due to a decrease in counts of plaque mutans streptococci and/or reduced amounts of extracellular polysaccharides in the plaque [18]. Also, the so-called xylitol-resistant mutans streptococci which were suggested to be easily shed to saliva have been connected with a decrease in the amount of plaque [18, 19].

Most of the xylitol studies showing a decrease in the amount of plaque have been conducted with chewing gum [16, 18]. Also, other sugar-free polyol gums may reduce plaque accumulation [20]. There is evidence that regular use of sugar-free chewing gum, in conjunction with normal oral hygiene, provides a small, but significant reduction in plaque [20, 21]. Sugar-free gum is recommended by several organizations, for example the American Dental Association (www.ada.org). However, to our knowledge, only one systematic review concerning sugar-free polyol gums and plaque accumulation has been published [20].

With this systematic review, we wanted to answer the defined research questions: (1) can the consumption of xylitol chewing gum or candies/lozenges/pastilles reduce the accumulation of dental plaque, (2) are the effects specific for xylitol? To achieve this, we described and evaluated the literature published during 1971–2020 in relation to the effect of xylitol chewing gums and candies on the amount of dental plaque in healthy children and adults.

## Materials and methods

The Preferred Reporting Items for Systematic Reviews and Meta-Analyses (PRISMA: www.prisma-statement.org) was used as a guideline in the present systematic review. The review was submitted for registration in PROSPERO on November 11, 2020 before the data collection started.

### Information sources for data extraction

A systematic review to identify all the relevant studies published was conducted from three databases: PubMed, Embase and the Cochrane Library. Grey literature was searched on www.clinicaltrials.gov. The searches were conducted on November 11, 2020 and updated on December 31, 2020.

### Literature screening strategies

The following terms were used in the search for studies:

- ('xylitol'/exp OR xylitol*) AND ('tooth plaque'/exp OR tooth NEXT/1 plaque* OR dental NEXT/1 plaque*)—Embase
- (xylitol* OR "Xylitol"[Mesh]) AND (dental plaque* OR "dental plaque"[Mesh] OR tooth plaque*)—PubMed
- (xylitol*) AND (dental NEXT plaque* OR tooth NEXT plaque*)—Cochrane Library

### Study inclusion and exclusion criteria

Prospective randomized controlled clinical trials (RCT) conducted in healthy subjects were included in the review. Only healthy subjects were included in the evaluation since if health claims are made on functional foods such as xylitol, the evaluated studies should be conducted in subjects who do not have problems with their general health. The aim of the included trials was to study the effects of xylitol on the amount of dental plaque. Chewing gums or candies (including lozenges/pastilles) were the vehicles included in the review. Plaque was either the primary or secondary outcome measure in the evaluated studies. The included studies compared baseline or no treatment values with values obtained in the same subjects after the intervention period. The comparison/control (product) was a polyol gum or candy, chewing gum base or no product. In order to meet the inclusion criteria, xylitol had to be the polyol with a concentration of 50% or more in the tested product. The comparison/control product could not contain xylitol.

Exclusion criteria used when evaluating abstracts: in vitro studies; animal studies; studies in subjects undergoing orthodontic treatment; studies in mentally retarded or disabled subjects; studies in children, elderly subjects or geriatric patients living in institutions; studies not related to oral health; reviews, abstracts, comments or study protocols; the polyol vehicles were oral rinses, toothpastes, oral sprays, pacifiers, milk or wipes; dental plaque was not an outcome of the study; other plaque reducers than xylitol were studied; no control group; mother-to-child transmission studies; the study was not available in English.

Exclusion criteria used when evaluating full-text articles: in five studies, baseline values were not available [22–26]; three studies were not properly controlled or the control did not fulfill the inclusion criteria [27–29]; two studies were cross-sectional [30, 31]; in one study, the test product contained less than 50% of all polyols [32]; one study was not randomized [33]; in one study, plaque was analyzed only at baseline [34]; and in one study, there was no information on the daily dose of xylitol [35].

### Data extraction and assessment of methodological quality and risk of bias

The articles that fulfilled the inclusion criteria were selected for full-text review and data extraction. The following data were collected: author and year of publication, study site, number and age of participants, study design, intervention and controls, oral hygiene instructions, assessment method, and main results.

The risk of bias of the selected articles was assessed using the Cochrane Collaboration tool for assessing risk of bias in randomized trials [36]. Two authors (ES, KP) independently evaluated the included abstracts and full-length articles and, based on mutual agreement, eliminated discrepancies between each individual assessment. A third evaluator (VL) evaluated the articles in which the first author of this review was an author.

The studies were appraised according to the following aspects: random sequence generation, allocation concealment, blinding, completeness of outcome data, selective reporting and funding bias. Each aspect was classified as having either low, high or unclear risk of bias. The bias was estimated to be unclear, for example, if the study was randomized but details on randomization were not given. Also, when information not found in the paper was submitted by the authors, the bias was classified as unclear. The overall level of risk for each study was classified as low (all quality items were met: high quality), unclear (unclear risk of bias for one or more domain: fair quality), or high (high risk of bias for one or more domain: low quality) [15, 36, 37].

## Results

### Study selection

**Fig. 1:**
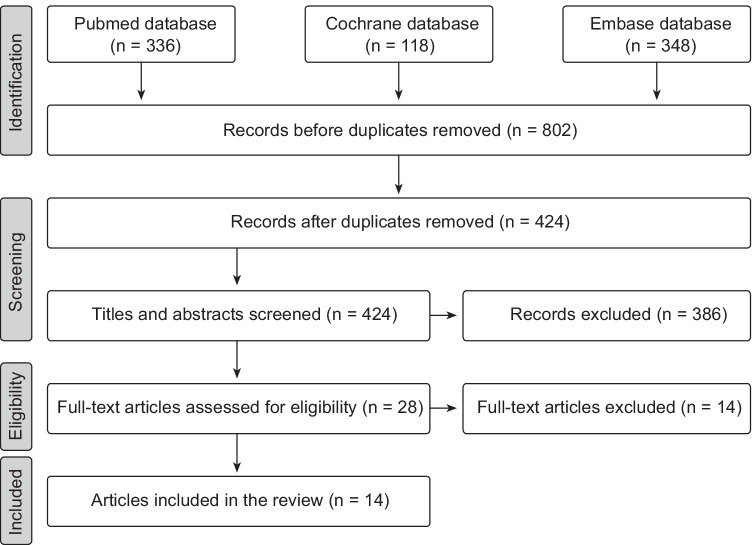
Flow chart

In the search for xylitol articles, a total of 802 titles were screened for relevance: (336 PubMed, 348 Embase, 118 Cochrane). Removing the duplicates left 424 titles to be evaluated. Based on the information of the abstract, 396 articles were removed. When full-text articles were assessed for eligibility, 14 articles were removed leaving 14 articles to be reviewed (Fig. 1). One of the articles consisted of three substudies, bringing the total number of evaluated studies to 16.

### Study characteristics

All studies included in the review were prospective, randomized, controlled studies published between 1971 and 2020 [38–51]. In the 16 studies included in the review, all participants were classified as healthy by the authors of the studies. All studies reported the age of the participants (age range 5–60 years or older), sample size (ranging from 14 to 485), and study duration (from 6 days to 3 years). The delivery modalities included chewing gums or candies (lozenges/pastilles). In four of the studies, the subjects were children (< 18 years); in 12 studies, the participants were adults (Table 1).

**Table 1:** Summary of the included studies

	Subjects; n	Study design; outcome measure	Intervention	Comparative	Assessment method	Results
Birkhed et al. (1979); Malmö, Sweden	19–58-yr-old adults, *n* = 110	Doubleblind, randomized, controlled study (3 mo); plaque amount (pom)	XYL lozenges (50%, 4 g/d, 4xd)	SOR, MAL, Lycasin lozenges (50%, 4 g/d, 4xd)	Fresh weight NOH 2 d	No decreases in the amount of plaque in any of the groups
Söderling et al. (1989); Ann Arbor, USA	19–35-old adults, *n* = 28	Blinded*, randomized*, controlled study (2 wk); plaque amount (pom)	XYL gum (76%, 11 g/d, 5xd), XYL-SOR gum (59% XYL, 17% SOR, 11 g/d, 5xd)	SOR gum (76%, 11 g/d, 5xd)	Fresh weight NOH 2 d CT not reported	Plaque decreased in the XYL and XYL-SOR groups, an increase in the SOR group (*p* < 0.05)
Steinberg et al. (1992); New York, USA	Adults, *n* = 28	Doubleblind, randomized, controlled cross-over study (6 wk); plaque amount (pom)	XYL gum (1.8 g/stick*, 9 g/d*, 5xd)	SOR gum (1.8 g/stick, 9 g/d, 5xd), no gum	Quigley-Hein PI NOH 0.5 d CT 5 × 10 min	The XYL (*p* < 0.001) and SOR (*p* < 0.05) gums decreased plaque compared to the baseline and no gum group
Cronin et al. (1994a); New Jersey, USA	> 18-yr-old adults, *n* = 59	Blinded, randomized, controlled study (2 wk); plaque amount (pom)	XYL-SOR gum (0.8 g XYL, 0.2 g SOR/piece, 8 g XYL/d, 5xd)	SOR gum (5xd)	Fresh weight NOH 2.5 d CT not reported	Plaque regrowth was reduced more by the XYL-SOR gum compared to the SOR gum (*p *< 0.05)
Cronin et al. (1994b); New Jersey, USA	> 18-yr-old adults, *n* = 154	Doubleblind, randomized, controlled trial (2 wk); plaque amount (pom)	XYL-SOR gum (0.8 g XYL, 0.2 g SOR/piece, 4.6–8 g/d, 3xd, 4xd, 5xd)	SOR gum (5xd)	Fresh weight NOH 2.5 d CT not reported	Plaque regrowth was reduced more by XYL-SOR gum compared to SOR gum (*p* < 0.05)
Cronin et al. (1994c); New Jersey, USA	> 18-yr-old adults, *n* = 142	Doubleblind, randomized, controlled study (2 wk); plaque amount (pom)	XYL pellet gum (850 mg/piece, 8.5 g/d, 5xd), XYL stick gum (850 mg/stick, 4.3 g/d, 5xd)	SOR gum (5xd)	Fresh weight NOH 2.5 d CT not reported	Plaque regrowth was reduced more by the XYL gums compared to the SOR gum (*p* < 0.01)
Merikallio et al. (1995); Pori, Finland	19–29-yr-old adults, *n *= 50 (MS > log 5)	Blinded*, randomized*, controlled study (2 wk); plaque amount (pom)	XYL gum (65%, 5.5–10 g/d, 3–5xd), XYL tablet (1.1 g, 3.3–5.5 g/d, 3–5xd)	No product	Fresh weight NOH 2 d CT not reported	No change in the amount of plaque in any of the groups
Tellefsen et al. (1996); Loma Linda, USA	21–35-yr-old adults, *n* = 14	Doubleblind, randomized, controlled cross-over study (6 d); plaque amount (pom)	XYL gum (0.8 g/piece, 4 g/d, 3xd)	SOR gum (1 g/piece, 5 g/d, 3xd)	Quigley-Hein PI NOH 6 d CT 3 × 20 min	XYL gum reduced plaque regrowth more than SOR gum (*p* < 0.01)
Söderling et al. (1997); Turku, Finland	23–25-yr-old adults, *n* = 37 (MS ≥ log 5)	Doubleblind, randomized*, controlled study (2 wk); plaque amount (pom)	XYL gum (65%, 6–10 g/d, 3–5xd), XYL-SOR gum (37.5% XYL, 37.5% SOR, 6–10 g/d, 3–5xd)	Gum base	Fresh weight NOH 2 d CT 3–5 × 3 min	XYL (*p* < 0.01) and XYL-SOR (*p* < 0.05) gums decreased plaque. No change in the gum base group
Mäkinen et al. (2005); Daegu, Korea	5-yr-old children, *n* = 149	Doubleblind, randomized, controlled trial (6 mo); plaque amount (pom)	XYL gum (80%, 4.5–5 g/d, 5xd)	SOR gum (73%, 4.5–5 g/d, 5xd), no gum	Quigley-Hein PI NOH not reported CT 4 × 5 min	XYL gum decreased plaque (*p* < 0.05), no change in the SOR gum group. Results for no gum not reported
Holgerson et al. (2007); Sävar, Sweden	7–12-yr-old children, *n* = 128	Doubleblind, randomized, controlled study (4 wk); plaque amount (pom)	XYL gum (77%, 6.2 g/d, 3xd)	SOR-MAL gum (SOR 64%, MAL 5%, 4.4 g/d, 3xd)	Simplified oral debris index NOH not reported CT 3 × 10 min	XYL and SOR/MAL gums decreased plaque compared to baseline (*p* < 0.05)
Al-Haboubi et al. (2012); London, UK	≥ 60-yr-old adults, *n* = 186	Doubleblind, randomized, controlled study (6 mo); plaque amount (som)	XYL gum (66%, 2.8 g/d, 2xd)	No gum	Silness-Löe PI NOH not reported CT 2 × 15 min	XYL gum decreased plaque (*p* < 0.001), no change in the no gum group
Runnel et al. (2013); Tartu, Estonia	7–8-year-old children, *n* = 485	Doubleblind, randomized, controlled trial (3 yr); plaque amount (som)	Xylitol candies (90%, daily dose appr. 7.5 g/d, 3xd)	SOR, ERY candies (90%, daily dose appr. 7.5 g, 3xd)	Fresh weight NOH 0.5 d	ERY candies decreased plaque (*p* < 0.05), no change in the XYL and SOR groups
Thabuis et al. (2013); YiXing, China	13–15-year-old children, *n* = 288	Doubleblind, randomized, controlled study (30 d); plaque amount (som)	XYL gum (59%, 10 g/d, 5xd)	MAL gum (59%, 10 g/d, 5xd), gum base, no gum	Quigley-Hein PI NOH 2 d CT 5 × 10 min	XYL and MAL gums decreased plaque compared to no gum (*p* < 0.05). Results for gum base not reported
Keukenmeester et al., (2015); Amsterdam, the Netherlands	> 18-yr-old adults, *n* = 303 (moderate gingivitis)	Doubleblind, randomized, controlled study (3 wk); plaque amount (som)	XYL gum (64%*, 9 g/day*, 5xd)	MAL gum (64%*, 9 g/day*, 5xd), gum base, no gum	Quigley-Hein PI NOH 0.5 d CT 5 × 10 min	XYL, MAL gums (*p* < 0.001) and gum base (*p* < 0.01) decreased plaque in brushed upper jaw. No changes in the no gum group or nonbrushed lower jaw
Akgül et al. (2020); Istanbul, Turkey	18–29-yr-old* adults, *n* = 154	Blinded, randomized, controlled study (3 wk); plaque amount (som)	XYL gum (5.4 g/d, 3xd)	Gum base*	Silness-Löe PI NOH not reported CT 3 × 10 min	XYL gum decreased plaque (*p* < 0.05), no change in the gum base group

*XYL*, xylitol; *SOR*, sorbitol; *MAL*, maltitol; *ERY*, erythritol; *PI*, plaque index; *MS*, mutans streptococci; *wk*, weeks; *yr*, years; *mo*, months; *d*, days; *min*, minutes; *pom*, primary outcome measure; *som*, secondary outcome measure; *NOH*, no oral hygiene; *CT*, recommended gum chewing time; *details on the study obtained from the authors

In the majority of the studies, the primary outcome measure was the amount of plaque (Table 1). In one study, the primary outcome measure was the acidogenicity of plaque [49], and in another, caries occurrence [48]. In one of the studies, the stimulated saliva flow rate was the primary outcome measure [47], in one, bleeding on marginal probing [50], and in one, pro-inflammatory cytokines [51].

### Quality assessment of the selected studies

**Fig. 2:**
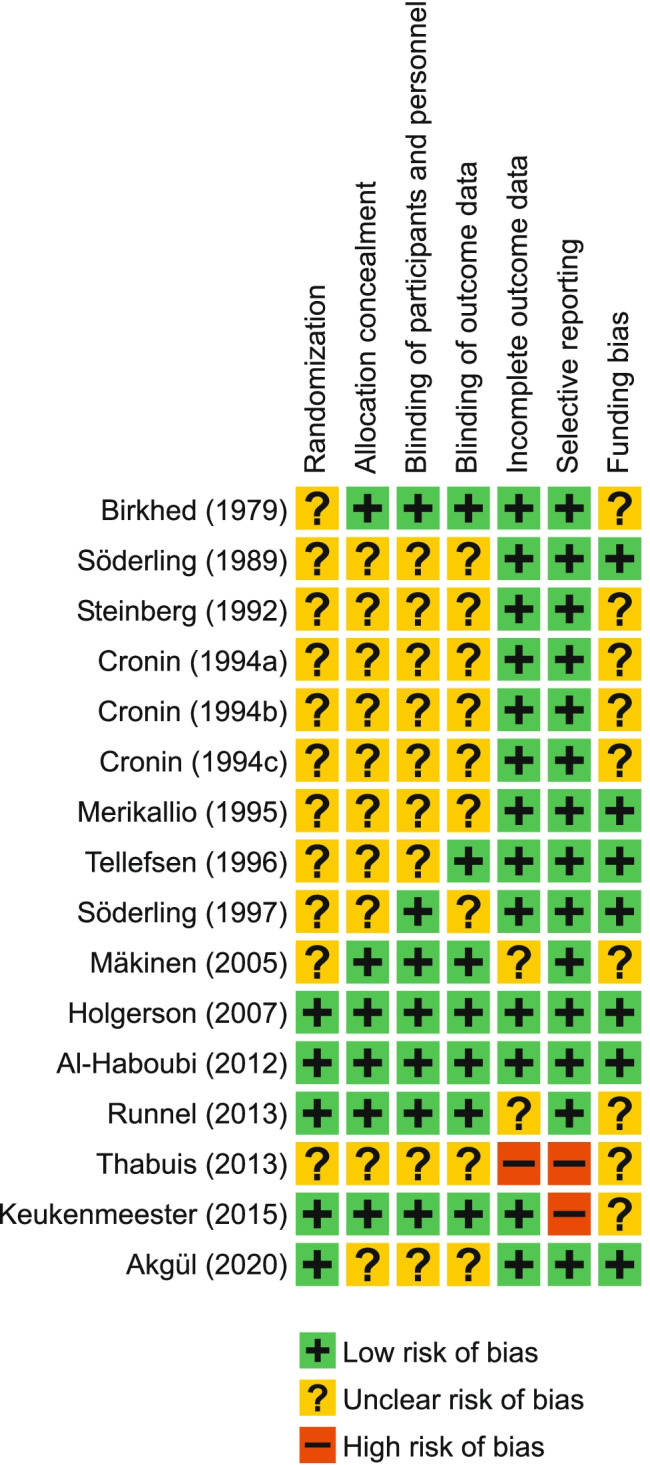
Risk of bias summary

Figure 2 summarizes the risks of bias in the evaluated studies. The risk-of-bias assessment revealed that two studies had a low risk of bias [46, 47], two studies [49, 50] were scored as having a high risk of bias, and the rest (12 studies) had an unclear risk of bias.

Since the present review includes studies from the 1970s, 1980s and 1990s, it is evident that details concerning for example randomization or blinding were lacking. In fact, only in four studies were the participants randomized on an individual basis using computer-generated randomization [46, 47, 50, 51]. In the study by Mäkinen et al. [45], the randomization was done according to kindergarten, which is a practical way to perform chewing gum studies, but may result in some bias.

The amount of plaque was the primary outcome of the present review, and thus it was important that the plaque collection or estimation of the plaque index were performed blinded. This important point was addressed only in a few studies; most studies merely included the statement that they were performed blinded, without providing much detail. However, problems with allocation concealment and blinding are inevitable when the control group does not chew gum or consume candies [42, 47].

The availability of individual baseline values increased the probability of finding true intervention-related changes in the amount of plaque in the evaluated studies. Also, differences in changes in the amount of plaque between intervention and comparison groups could be detected. In most studies, the baseline plaque values were comparable with the post-intervention values; however, in two studies, this point remained unclear [40, 43]. The crossover designs and controls of these studies should nevertheless compensate for the possible bias in the incomplete outcome data, thus presenting an unclear risk of bias [40, 43]. In the study by Runnel et al. [48], an unclear risk of bias in relation to incomplete outcome data was based on not taking the high number of dropouts into consideration.

Not including in the abstract a report of significant plaque reductions detected in the study led to a high risk of bias in relation to selective reporting in the study by Keukenmeester et al. [50]. In the study by Mäkinen et al. [45], results for the sorbitol control group were shown, but not for the no-gum control group leading to an unclear risk of bias in relation to incomplete outcome data. In the Thabuis et al. [49] study, the results for the gum-base control were not reported leading to a high risk of bias both in relation to incomplete outcome data and selective reporting.

In six of the studies, the tested xylitol and control products had been obtained as gifts from various companies without other apparent funding [39, 42, 44, 46, 47, 51]. Seven studies appeared partly or fully industry-funded resulting in and unclear risk of bias [38, 40, 43, 45, 48–50]. Also, the three separate clinical studies by Cronin et al. [41a, b, c] had an unclear risk of funding bias.

### Influence of xylitol chewing gum on the amount of plaque

Thirteen of the total of 14 chewing gum studies found a significant decrease in the amount of plaque in association with xylitol gum chewing [39–41a, b, c, 43–47,49–51]. The finding was similar in children [45, 46, 49] and adults [39, 40, 43, 44, 47, 50, 51]. Eleven of the studies were short-term studies lasting 6–30 days and three were long-term studies lasting 1.5–6 months.

In six of the studies, xylitol gum chewing decreased plaque accumulation compared to the sorbitol control gum [39, 43, 45]. This suggests specific effects for xylitol on plaque accumulation. In the 2-week American study, xylitol and xylitol-sorbitol chewing gum decreased the fresh weight of plaque by 24–29%, while an increase in plaque was seen in the sorbitol gum group [39]. The three 2-week studies by Cronin et al. [41a, b, c] compared, among other things, the effects of various daily doses of xylitol on fresh weights of plaque in association with consumption of xylitol/sorbitol and sorbitol gum. In the first study [41]a], plaque regrowth was reduced by 29% in the xylitol/sorbitol gum group, by 23–32% (consumption 2 pieces 3 day or higher) in the second study [41b] and by 32–38% in the third study [41c]. In the sorbitol control groups, the plaque regrowth reductions were small, 8–10% [41a, b, c]. In the 6-month study by Mäkinen et al. [45], xylitol gum chewing four times a day, 5 min at a time, decreased the mean plaque index of 5-year-old children by 8% while no change was seen in the sorbitol control group. The chewing gum study by Tellefsen et al. [43] compared plaque accumulation using a plaque index after 6 days of no oral hygiene combined with xylitol or sorbitol gum chewing, 3 × 20 min a day, xylitol gum reducing plaque formation more than the sorbitol gum.

In four studies, both the xylitol gum and the control polyol gums showed similar decreases in plaque accumulation [40, 46, 49, 50]. In all of these studies, the gum chewing recommendation was 3–5 times a day, 10 min at a time. In the study by Steinberg et al. [40], 1.5 months of chewing xylitol or sorbitol gum decreased the mean plaque index by 15% in the xylitol group, and by 12% in the sorbitol control group, while in the no-gum group, no decrease was detected. In the 4-week, Swedish study, the plaque index measured from six buccal surfaces decreased both in the xylitol and sorbitol gum group [46]. In two low-quality studies, the xylitol gum and the controls, maltitol gum [49] or maltitol gum and gum base [50], decreased the plaque index. In the 30-day study by Thabuis et al. [49], a plaque index decrease of 43% was reported both in the xylitol and maltitol groups. In the Keukenmeester et al. [50] 3-week study, a decrease in the plaque index of 7–11% was found in the brushed upper jaw in the xylitol and maltitol groups. However, in the absence of brushing, no differences were detected in any group in plaque accumulation in the lower jaw.

Gum base was used as a control in four xylitol chewing gum studies [44, 49–51], but results were reported only in three studies [44, 49, 51]. In two studies, it was the only control [44, 51]. In a Finnish study, xylitol and xylitol-sorbitol gums decreased the fresh weight of plaque by 32–34% while the gum base comparison did not [44]. In the study, the gums were recommended to be chewed 3–5 times a day, 3 min at a time. In the recent study by Akgül et al. [51], xylitol gum chewing decreased plaque accumulation by 46% while a small reduction of 9% was seen in the gum base group. The recommended chewing time was 3 times a day, 10 min at a time. In the Keukenmeester et al. study [50], similar small decreases in the amount of plaque were observed in the xylitol and gum base groups. In the high-quality Al-Haboubi et al. [47] study, the comparison of xylitol gum was no gum. The low mean plaque scores of elderly people further decreased in association with xylitol gum chewing for 6 months, twice a day, for 15 min at a time. The plaque index did not change in the no-gum control group.

The only chewing gum study not demonstrating a plaque decrease for xylitol gum was a 2-week study comparing the effects of xylitol gum and army-made pastilles with no gum [42]. The very high plaque amounts suggest that the recruits omitted oral hygiene totally in field conditions.

### Influence of xylitol candies/pastilles on the amount of plaque

In three studies, the consumption of xylitol candies/lozenges/pastilles showed no effect on plaque accumulation [38, 42, 48]. In the study by Birkhed et al. [38], the subjects consumed xylitol, sorbitol, maltitol and Lycasin lozenges for 3 months. The mean fresh weights of plaque did not decrease in any of the groups. In the 3-year study by Runnel et al. [48], the effects of xylitol, erythritol and sorbitol candies on plaque accumulation were compared. Erythritol candies appeared to decrease plaque, while no effect was seen for either xylitol or sorbitol candies.

### Adverse effects

Possible adverse effects connected with the use of the test and control products were recorded and reported in five of the 16 studies [41c, 45,47,49,50. One subject discontinued the study based on feeling nauseous due to gum chewing [41c], no other adverse effects were reported in the five studies.

## Discussion

The main finding of this review is that habitual xylitol chewing gum consumption reduces the amount of plaque. The result appeared to be similar in short- and long-term studies, and in children and adults. In all but one study [47], the daily doses of xylitol were high enough, approximately 5 g or higher, to achieve “xylitol effects” demonstrated, for example, in association with decreases in counts of mutans streptococci [15, 52]. In six studies, xylitol gum chewing decreased plaque compared to sorbitol gum suggesting specific effects for xylitol on plaque accumulation [39, 41a, b, c, 43,45].

It has been suggested that chewing gum has no relevant tooth-cleaning effects [10]; however, a small, but significant plaque-reducing effect has been shown for sugarfree chewing gums in several studies [20]. Polyols like sorbitol and maltitol are used commonly as controls in chewing gum studies. They are considered microbiologically rather inert but they are sweet and thus add to the saliva secretion-enhancing effect of gum base chewing. Gum base is often a problematic control in the studies, since without the polyol sweetener, the gum may be harder to chew and the taste is not as agreeable as in the polyol-containing gums. In three of the evaluated studies, results for a control gum base were reported [44, 49, 51]. In our evaluation, gum base chewing showed no [44] or small 7–9% decreases [50, 51] in the mean plaque indices. Thus, the plaque-reducing effects of polyol chewing gums may not be attributed to chewing per se.

Our results suggest that chewing time may be a confounding factor in chewing gum studies even though all evaluated studies did not support this idea. In six studies with short chewing time recommendations [44, 45] or no recommendations [39, 41a, b, c], the xylitol gum decreased plaque while sorbitol gum/gum base did not, suggesting that xylitol gum exerts specific plaque-reducing effects. Xylitol dissolves from a chewing gum with a high concentration peak in the saliva at 1 min, the bulk of the xylitol (and sweetness) being dissolved at 3 min of gum chewing [53]. Thus, if no chewing time recommendations are given, the tasteless gum is most probably discarded after a short chewing period. The short chewing time resulting in high xylitol levels in the plaque may be important for the mechanism of action of xylitol. Extended gum chewing may actually decrease the beneficial effects of xylitol by stimulating the salivary flow for a long time and thus promoting oral clearance of xylitol. This may be crucial for the effects of xylitol chewing gum on plaque. In fact, the longer chewing recommendations, 3–5 × 10 min, were in three studies associated with similar small plaque reductions for xylitol, sorbitol and maltitol gums [40, 46, 50]. This result is in accordance with earlier studies with sorbitol gum [40, 41, 43–45, 54–56]. In these studies, the sorbitol gum was chewed several times a day, 20–30 min at a time, and the plaque decreases were significant but rather small. Since the chewing time may influence the outcome of chewing gum studies, it could be an interesting research aspect of future chewing gum studies.

The three xylitol studies with candies/lozenges/pastilles did not find effects for xylitol on the amount plaque [38, 42, 48]. In the study by Birkhed et al. [38], the subjects showed high amounts of plaque compared to similar studies with 2 days of no oral hygiene [39, 44], which may be a confounding factor. Also, the intervention lasted only 4 days and the daily dose of xylitol was rather low, 4 g, which may have affected the outcome of the study. In the Runnel et al. [48] study, the authors postulated that treatment during the span of the study was relatively mild: test products were only consumed three times a day with the last consumption around 2 pm, and the products were consumed during weekdays, and not during the school vacation. Studies which did not fulfill the inclusion criteria of the present review have, however, demonstrated plaque decreases for xylitol candies/pastilles. Significant plaque reductions have been observed in association with consumption of xylitol candy and pastilles in disabled Finnish [57, 58] and Kuwaitian subjects [59].

Xylitol may influence plaque accumulation through several mechanisms. Xylitol consumption has reduced the acid production potential of plaque [60], thus not favouring acidogenic and aciduric microorganisms like mutans streptococci. There is good evidence suggesting that habitual xylitol consumption reduces mutans streptococci counts in plaque [15], which could result in less adhesive plaque. In our evaluation, in one fair-quality study, associations between the magnitudes of the decreases in the fresh weights of plaque vs. levels of both mutans streptococci and xylitol-resistant mutans streptococci were analyzed, but no associations were detected [44]. It has also been suggested that a xylitol-induced decrease in the extracellular polysaccharides could reduce plaque [61]. In one study, no connection was detected between decreases in the plaque fresh weights and the polysaccharide contents of plaque [44]. However, in the low-quality study of Thabuis et al. [49], insoluble glucans of plaque were reported to decrease in the xylitol and maltitol gum groups but not in the no-gum or gum-base groups. Clearly, there is a need for more research on this topic.

We included in the present evaluation studies with individual baseline values available. The baseline values clearly increased the transparency of the studies and were usually associated with relevant statistical testing. Also, studies with relatively low numbers of subjects could give relevant results concerning changes in the amount of plaque. If the methods were valid, even old studies could be considered to be of at least fair quality. In addition to the study design, also other methodological aspects are of importance, especially in plaque studies. Eight of the present studies estimated the amount of plaque with gravimetric methods [38, 39, 41a, b, c, 42,44,48] and seven used plaque indices. The plaque indices used were Quigley-Hein or its modification [40, 43, 45, 49, 50], Silness-Löe [47, 51] or a simplified oral index (OHI-S; 46). In most of these studies, plaque indices were presented as means of plaque scores [40, 43, 45, 47, 49–51]. Since plaque is not accumulated evenly in the mouth and the index values are categorical in nature, the means of the scores may not reflect properly plaque accumulation. Also, plaque indices do not take into account, for example, the thickness of plaque. In two studies, no oral hygiene was practiced for 6 days [43] and 3 weeks [50], but this was poorly reflected in the mean scores of plaque. As for the Holgerson et al. [46] study, as the authors themselves state, the simplified oral debris index may not be an adequate way to quantify plaque. Gravimetric methods should be more accurate compared to plaque indices, especially when mean scores are calculated and/or only a few index teeth were used for estimating the plaque index. This idea is supported by a study by Birkhed et al. [25] which compared plaque indices and gravimetric methods in an intervention study.

Also, the recommendations concerning no oral hygiene before the plaque collections should be of importance. The study subjects will not omit oral hygiene if no instructions are given, and the effects of the interventions may be difficult to detect if there is very little plaque. Our results support this idea. In subjects adhering to 2–2.5 days of no oral hygiene before the plaque collections, clinically relevant changes in the amount of plaque, approximately 20–40%, were detected for xylitol gum chewing [39, 41a, b, c, 44,49]. If no recommendations were given or oral hygiene was omitted only in the morning of the plaque collection date, the changes in the amount of plaque were, as a rule, small [40, 45, 50]. In two studies, the subjects refrained from all oral hygiene measures during the study for 6 days or 3 weeks [43, 50]. In these studies, xylitol gum chewing reduced plaque accumulation compared to sorbitol gum [43] or had no effect on it [50]. In the 2-week study conducted in the army, no effects of xylitol gum chewing or consumption of xylitol pastilles were detected. Based on the very high amounts of plaque, the recruits apparently did not follow the 2-day no-oral-hygiene recommendation but omitted toothbrushing totally during the study [42]. It is clear that xylitol gum is no substitute for toothbrushing.

Adverse effects were registered in 5 out of the 16 reviewed studies [41, 45, 47, 49, 50], and reported only for one subject who discontinued the study based on feeling nauseous when chewing gum [41]. In three studies, one of high and two of low quality [46, 49, 50], the chewing gums were chewed 3–5 times a day, and the recommended chewing times were rather long, 10 min at a time. In two studies, the recommended chewing times were even longer, 15/20 min [43, 47]. Thus, it may be expected that the subjects would experience flatulence or have problems with temporomandibular joint dysfunction, especially if they were older [47], but this was not the case. Also, the polyol sweetener could have been associated with adverse effects. Xylitol, sorbitol and maltitol belong to FODMAP (Fermentable Oligo-, Di-, Mono-saccharides And Polyols) substances which may not suit persons with a tendency for digestive disorders. No such adverse effects connected with the polyol sweeteners were reported in the evaluated studies. In fact, complaints about digestive discomfort in xylitol studies are rare [15, 62].

Effects of xylitol and other polyols on plaque accumulation have received little interest after 2000. Only seven studies published between 2005 and 2020, two of them of low quality, fulfilled our inclusion criteria. Recently, Indian research groups have published results on the effects of xylitol on the amount of plaque [35, 63]. These papers did not meet the inclusion criteria of the present review. Xylitol could, however, especially in chewing gums, be an important adjunct to routine oral hygiene methods such as toothbrushing [64]. A reduction in dental plaque formation would benefit subjects of all ages and health conditions. Improving oral hygiene is an important issue for example in elderly people, who would benefit from an agreeable way to decrease plaque formation. In the high-quality study by Al-Haboubi et al. [47], older people living in the community chewed xylitol gum for 6 months and showed improvement in plaque indices, gingival scores and also self-perceived oral health [47]. Most studies published on the effects of xylitol on plaque accumulation report results on caries-associated variables like levels of mutans streptococci and/or the acid production potential of plaque [38, 39, 45, 46, 48, 49]. Only a few studies, however, have been published on the effects of xylitol on the composition of the microbiota reflecting the resistance of the microbiota to dysbiosis [15]. There is also a demand for studies on the impact of polyol products on gingival health. Of the evaluated papers, in addition to the study by Al-Haboubi et al. [47], only three studies dealt with the effects of xylitol gum chewing on gingival health [40, 50, 51].

To our knowledge, our systematic review is the first review to deal with the effects of xylitol consumption on plaque accumulation. Seven of the present xylitol chewing gum articles were conducted in adults, and thus could have been evaluated in the Keukenmeester et al. [20] systematic review on the effects of sugar-free gum on plaque. However, only the old study by Steinberg et al. [40] is included in both our and the Keukenmeester et al. [20] review. The review evaluated three xylitol gum studies from the 1970s; these studies did not fulfill our inclusion criteria. Two of them had no baseline values [22, 23] making proper interpretation of the results difficult and in one, the amount of plaque was not an outcome measure [65].

We found 16 studies that met the inclusion criteria of the review. Surprisingly, considering that most of the studies were rather old, 14 showed a high or fair quality. A meta-analysis or high-detailed scoring might have improved the review [66]. The studies were, however, very heterogeneous with respect to subjects, methods and study designs making a meta-analysis difficult to perform and interpret. Regardless, it is a strength of the review that the included studies compared baseline or no treatment values with values obtained after the intervention period decreasing a possible risk of bias. The review also takes into consideration methodological issues, which are often overlooked.

The present review identified 12 of the altogether 14 xylitol chewing gum studies with either high or fair quality. Based on their results, it is likely that habitual use of xylitol chewing gum decreases plaque in children and adults both in short-term and long-term consumption. The plaque-reducing effects of xylitol gum also appear to differ from those of sorbitol gum/gum base. The recommended chewing time may be a confounding factor in chewing gum studies. Based on three fair quality studies, xylitol consumption of candies/lozenges/pastilles appears not to decrease plaque accumulation. Effects of xylitol consumption on plaque accumulation and its clinical impact clearly need further, well-controlled studies.
